# Classification and Statistical Trend Analysis in Detecting Glaucomatous Visual Field Progression

**DOI:** 10.1155/2019/1583260

**Published:** 2019-05-28

**Authors:** Cristiana Valente, Elisa D'Alessandro, Michele Iester

**Affiliations:** Anatomical-Clinical Laboratory for Functional Diagnosis and Treatment of Glaucoma and Neuro-ophthalmology, Eye Clinic, DiNOGMI, University of Genoa, IRCCS Ospedale Policinico San Martino, Genoa, Viale Benedetto XV 5, 16132 Genoa, Italy

## Abstract

**Aim:**

To evaluate the agreement between different methods in detection of glaucomatous visual field progression using two classification-based methods and four statistical approaches based on trend analysis.

**Methods:**

This is a retrospective and longitudinal study. Twenty Caucasian patients (mean age 73.8 ± 13.43 years) with open-angle glaucoma were recruited in the study. Each visual field was assessed by Humphrey Field Analyzer, program SITA standard 30-2 or 24-2 (Carl Zeiss Meditec, Inc., Dublin, CA). Full threshold strategy was also accepted for baseline tests. Progression was analyzed by using Hodapp–Parrish–Anderson classification and the Advanced Glaucoma Intervention Study visual field defect score. For the statistical analysis, linear regression (*r*^2^) was calculated for mean deviation (MD), pattern standard deviation (PSD), and visual field index (VFI), and when it was significant, each series of visual field was considered progressive. We also used Progressor to look for a significant progression of each visual field series. The agreement between methods, based on statistical analysis and classification, was evaluated using a weighted kappa statistic.

**Results:**

Thirty-eight visual field series were analyzed. The mean follow-up time was 6.2 ± 1.53 years (mean ± standard deviation). At baseline, the mean MD was −7.34 ± 7.18 dB; at the end of the follow-up, the mean MD was −9.25 ± 8.65 dB; this difference was statistically significant (*p* < 0.001). The agreement to detect progression was fair between all methods based on statistical analysis and classification except for PSD *r*^2^. A substantial agreement (*κ* = 0.698 ± 0.126) was found between MD *r*^2^ and VFI *r*^2^. With the use of all the statistical analysis, there was a better time-saving.

**Conclusions:**

The best agreement to detect progression was found between MD *r*^2^ and VFI *r*^2^. VFI *r*^2^ showed the best agreement with all the other methods. GPA2 can help ophthalmologists to detect glaucoma progression and to help in treatment decisions. PSD *r*^2^ was the worse method to detect progression.

## 1. Introduction

Glaucoma is a chronic disease characterized by an optic neuropathy with irreversible damage to the optic nerve head (ONH) and visual field (VF). From population studies, we know that increased intraocular pressure is associated with increased prevalence [1, 2] and incidence [3] of glaucoma. With increasing life expectancy, it is fundamental to slow down its progression to avoid visual disability and blindness. Unfortunately, often times, the disease progresses despite treatment; for this reason, it is important to monitor changes and in particularly rates of change. These changes may be observed by analyzing the ONH and the VF [4]. It is not easy to recognize VF changes due to test variability [5–7], even if field series are evaluated by experienced observers. Many algorithms are able to distinguish between fluctuation of sensitivity and progression, but no single one has been identified as being superior to the other [8–10].

There are many approaches for detecting and quantifying clinical progression [11]. Most often, progression is based on the comparison of serial visual field printouts by expert clinicians. However, this is often insufficient to reliably determine progression; for this reason, classification and statistical approaches exist. Classification systems such as Hodapp–Parrish–Anderson [12] classification and the Advanced Glaucoma Intervention Study [8] (AGIS) systematically score research protocols evaluating both changes in depth and location (cluster analysis). These methods are time-consuming but less subjective. The statistical methods are based on event and trend analysis. While the event analysis looks for the subjective identification of a confirmed event of change in a visual field series compared to a baseline exam, the trend analysis uses linear regression of the VF indices or of the sensitivity of the tested points to detect glaucoma progression over time.

The purpose of this study was to examine the level of agreement between classification systems (Hodapp et al. [12], AGIS [8]) and trend statistical methods (linear regression of MD and PSD, Humphrey Guided Progression Analysis (GPA) 2, and Progressor) in assessing VF progression.

## 2. Patients and Methods

This was a retrospective and longitudinal study with at least 5 years of follow-up (F/U). We followed in part the methods of Iester et al. [4]. The study, made in agreement with the tenets of the Declaration of Helsinki, included 20 Caucasian patients (mean age 73.8 ± 13.43 years) with primary open-angle glaucoma recruited from the MI's Glaucoma Clinic. Visual fields were assessed by Humphrey Field Analyzer 750 II, (HFA, Carl Zeiss Meditec, Dublin, California, USA), using the 30-2 or 24-2 SITA standard (Swedish interactive thresholding algorithm) test. The first 3 fields in each series were excluded to minimize learning effects: the forth and fifth full threshold or SITA Standard exams were used as baseline. Full threshold strategy was also accepted only for baseline tests. Patients were classified as having primary open-angle glaucoma when they had a typical abnormal ONH and/or a typical glaucomatous VF, open angle at gonioscopy, IOP>21 mm·Hg before treatment, and no clinically apparent secondary cause for their glaucoma [13]. The abnormal ONH classification [14] was based on the presence of an optic rim notch or of diffuse/generalized loss of optic rim tissue, vertical cup/disc diameter ratio asymmetry unexplained by differences in optic disc size, or disc hemorrhage. A glaucomatous VF defect [14] was defined as three adjacent points depressed by 5 dB, with one of the points depressed by at least 10 dB and two adjacent points depressed by 10 dB, or a 10 dB difference across the nasal horizontal meridian in two adjacent points. None of the points could be edge points unless immediately above or below the nasal horizontal meridian. In addition, visual field testing was considered reliable only when false-negative responses and fixation losses were less than 20%; unreliable VFs were not included in the analyses. Mean deviation (MD) and pattern standard deviation (PSD) were considered in the study to describe the included patients. Included patients presented with a typical glaucomatous visual field (baseline MD>3). Exclusion criteria were concomitant ocular disease (for example, cataract), previous ocular surgery, systemic disease or medication known to affect the VF, refractive error exceeding 8D spherical equivalent or 3D of astigmatism, and visual acuity < 20/50 at baseline or during the F/U.

Progression was analyzed by using classification systems and statistical analysis.

### 2.1. Hodapp–Parrish–Anderson Classification

This classification is based on two criteria. The first criterion is the overall extent of damage, which is calculated by using both the MD value and the number of defective points in the Humphrey Statpac 2 pattern deviation probability map of the full threshold test. The second criterion is based on the defect proximity to the fixation point. This system divides early, moderate, and severe glaucomatous visual defects [13] and recognizes the progression of visual field glaucoma damage if a new defect in a previously normal area or a decrease of sensitivity of a previously defect or a previously defect that became larger or a general depression of visual field sensitivity appears [12].

### 2.2. Advanced Glaucoma Intervention Study Scoring System

It is a quantitative method used to assess test reliability and to measure visual field defect severity using the Humphrey threshold test. The AGIS visual field defect score is based on both the number and depth of adjacent depressed test locations in the nasal area, upper hemifield, and lower hemifield. This score is obtained from the total deviation plot of the Statpac 2 single field analysis. A point is considered to be defective when a minimum amount of sensitivity depression is reached. Scores for each hemifield and for the nasal area are summed. The maximum possible score is 20 (two for the nasal field and nine for each hemifield). Progression is defined as an increase in score by 4 or more in three consecutive follow-up fields [15].

### 2.3. Guided Progression Analysis 2 (GPA2)

The visual field index (VFI) [16] is the trend analysis algorithm included in the GPA2. The VFI is a global parameter adjusted for age and expressed in percentage (a perimetrically normal field is set at 100% and a perimetrically blind one at 0%). To decrease the influence of cataracts, the pattern deviation probability map is used to identify test points with normal sensitivity (considered normal and scored 100%), those showing relative loss (these are scored as a function of total deviation and age-corrected normal threshold) and those showing no sensitivity (scored 0%). The VFI implements a weighting procedure that assigns more importance to central points than to peripheral points (considering 5 concentric rings of increasing eccentricity in the visual field plot). A significant trend was considered when the change in the VFI slope was considered as statistically significant (*p* < 0.05) by GPA2. A minimum of five exams over 3 years must be included in GPA2 for the linear regression results to be presented. The length of projection is equal to the number of years of GPA2 data that is available, up to a maximum projection time of 5 years.

### 2.4. Progressor

It performs a point-by-point linear regression analysis of sensitivity on time for the whole visual field series. The program produces a cumulative graphical output of each test location over time, using bar graphs. The height of the bar graphs above or below a horizontal line represents the sensitivity of the testing location above or below 30 dB, respectively, allowing visual comparison of each point over time by the height of the subsequent bars. Significant rate of changes is shown through color coding. Pointwise linear regression analysis requires at least two fields to generate a slope and a minimum of five fields to be clinically useful. The pointwise linear model has been demonstrated to provide a valid framework for detecting and forecasting glaucomatous loss [10]. In this study, the level of statistical significance we used was *p* < 0.05, and the rate of decibel loss we considered clinically significant was >1 dB/year.

### 2.5. Mean Deviation (MD)

It gives an overall value of the total amount of visual field loss, with normal values typically within 0 dB to −2 dB.

### 2.6. Pattern Standard Deviation (PSD)

It measures irregularity by summing the absolute value of the difference between the threshold value for each point and the average visual field sensitivity at each point.

### 2.7. Statistical Analysis

For the statistical analysis, linear regression (*r*^2^) was obtained from GPA2 (VFI) and Progressor, and *r*^2^ was calculated for MD and PSD. When significant (*p* < 0.05), each VF series was considered progressive.

The agreement between methods, based on statistical analysis and classification, was evaluated using a weighted kappa statistic. The *κ* statistic interpretation was as follows: *κ* < 0, no agreement; *κ* = 0.0 to 0.19, poor; *κ* = 0.20 to 0.39, fair; *κ* = 0.40 to 0.59, moderate; *κ* = 0.60 to 0.79, substantial; and *κ* = 0.80 to 1.0, almost perfect agreement.

Time to decide if the VF series were getting worse was calculated for each method.

## 3. Results

A total number of 303 visual fields divided into 38 VF series of 20 patients were analyzed. Visual field tests were not possible to perform in two eyes for the low visual acuity. When possible, in the study, both eyes' visual fields were analyzed for patients, and the data were analyzed independently in a masked way without knowing the other eye status.

The mean follow-up time was 6.2 ± 1.53 years (mean ± standard deviation). At baseline, the mean MD was −7.34 ± 7.18 dB and the mean PSD was 5.67 ± 4.09 dB. At the end of the follow-up, the mean MD was −9.25 ± 8.65 dB and the mean PSD was 6.92 ± 4.67 dB. The difference in perimetric indices at baseline compared to those at end of follow-up were statistically significant (Student's *t*-test *p* < 0.05 and *p* < 0.001, respectively, for MD and PSD).

Among the 38 VF series, 21 were considered as progressing using the Hodapp classification, 11 using the AGIS scoring system, 13 with MD *r*^2^, 5 with PSD *r*^2^, 12 with VFI *r*^2^, and 13 with Progressor. The agreement in detecting progression was ≥ *κ* = fair for all methods except for PSD *r*^2^. A substantial agreement (*κ* = 0.698 ± 0.126) was found between MD *r*^2^ and VFI *r*^2^ (Table 1).

The mean time, expressed in minutes, needed to evaluate the progression of VF series using the different methods is summarized in Table 2. The difference in time between classification systems (Hodapp et al. [12], AGIS [8]) and statistical methods (MD *r*^2^, PSD *r*^2^, VFI *r*^2^, and Progressor) was statistically significant (Wilcoxon/Mann–Whitney test; *p* < 0.001) with statistical methods being less time-consuming.

## 4. Discussion

The ability to detect the progression of visual field defects remains one of the most challenging aspects of glaucoma management. We found that, with the use of all the statistical analyses, there was a greater time-saving which is very important in the clinical practice [17]. In the event-based statistical approach [18], the criterion for progression is defined at the start of the study, and progression is confirmed when changes in VF have dipped below the preset threshold. Information at baseline and that from the most recent test is used to decide whether an eye has progressed or not. Trend-based statistical methods [18] can be adopted to improve progression rate measurement. These may be applied to the MD or VF sectors or at individual test locations over time by linear regression analysis. These approaches have been shown to be more sensitive in detecting progression than event-based analysis because all VF measurements over the course of follow-up are taken into consideration for the analysis; however, it is less accurate to inform on the location of the damage.

In our study, the Hodapp et al. [12] classification identified a greater number of progressive VF series compared to the other methods. Hodapp et al. [12] classification can be of great use in deciding when to start treatment once glaucoma has been diagnosed and how aggressive therapy should be, which is usually based on individual visual defect severity. The disadvantages include the fact that this three-stage subdivision is too simplified and thus may make it inappropriate for a fine categorization of visual field defects. Moreover, it requires an accurate and time-consuming analysis of every single visual field test result [19].

We can explain the poor agreement found between PSD *r*^2^ and all the other methods as the PSD analyzes localized VF defects. For this reason, it is not a good index when it is used in the POAG follow-up. In fact while the PSD is able to detect localized defects in early glaucoma, it is completely insensitive to a decline in the global background visual field level. PSD improves in the advanced stage of the disease; thus, it is not useful in the follow-up when the damage is advanced.

In our study, four eyes (11%) were identified as progressing by all statistical methods except PSD *r*^2^. This low concordance amongst different techniques has also been reported by other investigators [20, 21].

Up to now, many methods have been developed for assessing glaucomatous VF progression, but there is no gold standard method to detect progression. These methods that quantify the rate of visual field progression seem, however, to be the most appropriate for guiding treatment decisions [22].

In our study, VFI *r*^2^ showed the best agreement with all other methods, especially with MD *r*^2^. The substantial agreement found between VFI *r*^2^ and MD *r*^2^ is because both are statistical reductions of the visual field sensitivity. VFI was built considering MD values corrected with some coefficients calculated on the position of the tested points: the paracentral points weigh more heavily in VFI than the more peripheral ones [16]. Furthermore, VFI expresses the field as a percentage of the “normal,” whereas MD in dB scale. The MD makes no such assumptions, so is physiologically more robust. VFI is less affected by cataract and other media changes than MD when using the pattern deviation probability map. It allows a quantification of the VF series by comparing the defect depth with the normal age-adjusted visual field. This is done by taking into account the functional damage related to eccentricity to correlate with ganglion cell density. Moreover, it shows field status as a percentage. The latter easily allows, even for an ophthalmologist not specialized in glaucoma, to determine the rate of VF progression, therefore allowing to set a target intraocular pressure and instilling an individualized treatment. Furthermore, in the literature, different opinions about the virtues of the VFI over a long-established compared to MD are still present and on study [16, 23–26].

In conclusion, the best agreement to detect progression was found between MD *r*^2^ and VFI *r*^2^, while PSD *r*^2^ was the worse method to detect progression. VFI *r*^2^ showed the best agreement with all the considered event analysis methods. GPA2 could help ophthalmologists to detect glaucoma progression and to help in treatment decisions because of the VFI analysis and the event analysis graph which could help to identify the VF area where the changes occur.

## Figures and Tables

**Table 1:** Kappa statistic (k) among different methods to classify visual field progression.

	K	SE	IC 95%
*Hodapp et al. [12]*			
AGIS [8]	0.496	0.137	0.228; 0.764
Progressor	0.592	0.128	0.342; 0.843
MD *r*^2^	0.389	0.146	0.103; 0.675
PSD *r*^2^	0.121	0.15	−0.174; 0.415
VFI *r*^2^	0.432	0.143	0.151; 0.714

*AGIS [8]*			
Hodapp et al. [12]	0.496	0.137	0.228; 0.764
Progressor	0.636	0.136	0.368; 0.903
MD *r*^2^	0.514	0.153	0.215; 0.813
PSD *r*^2^	0.084	0.219	−0.344; 0.513
VFI *r*^2^	0.559	0.15	0.264; 0.853

*MD r* ^*2*^			
Hodapp et al. [12]	0.389	0.146	0.103; 0.675
AGIS [8]	0.514	0.153	0.215; 0.813
Progressor	0.298	0.168	−0.030; 0.627
PSD *r*^2^	0.177	0.197	−0.208; 0.562
VFI *r*^2^	0.698	0.126	0.452; 0.944

*PSD r* ^*2*^			
Hodapp et al. [12]	0.121	0.15	−0.174; 0.415
AGIS [8]	0.084	0.219	−0.344; 0.513
Progressor	−0.097	0.209	−0.507; 0.312
MD *r*^2^	0.177	0.197	−0.208; 0.562
VFI *r*^2^	0.2	0.202	−0.196; 0.597

*VFI r* ^*2*^			
Hodapp et al. [12]	0.432	0.143	0.151; 0.714
AGIS [8]	0.559	0.15	0.264; 0.853
Progressor	0.336	0.168	0.007; 0.665
MD *r*^2^	0.698	0.126	0.452; 0.944
PSD *r*^2^	0.2	0.202	−0.196; 0.597

*Progressor*			
Hodapp et al. [12]	0.592	0.128	0.342; 0.843
AGIS [8]	0.636	0.136	0.368; 0.903
MD *r*^2^	0.298	0.168	−0.030; 0.627
PSD *r*^2^	−0.097	0.209	−0.507; 0.312
VFI *r*^2^	0.336	0.168	0.007; 0.665

SE: standard error, IC: interval confidence, MD *r*^2^: mean deviation linear regression, PSD *r*^2^: pattern standard deviation linear regression, and VFI *r*^2^: visual field index linear regression.

**Table 2:** The mean time in minute needed to evaluate the progression of VF series using the different methods.

	Mean minutes ± SD
Hodapp et al. [12]	16.6 ± 7.7
AGIS [8]	17.3 ± 11
MD/PSD/VFI *r*^2^	5.2 ± 0.5
Progressor	1.6 ± 0.7

## Data Availability

The data used to support the findings of this study are available from the corresponding author upon request.
